# Plasticity and associated epigenetic mechanisms play a role in thermal evolution during range expansion

**DOI:** 10.1093/evlett/qrac007

**Published:** 2023-01-31

**Authors:** Janne Swaegers, Simon De Cupere, Noah Gaens, Lesley T Lancaster, José A Carbonell, Rosa A Sánchez Guillén, Robby Stoks

**Affiliations:** Laboratory of Evolutionary Stress Ecology and Ecotoxicology, University of Leuven, Leuven, Belgium; Laboratory of Evolutionary Stress Ecology and Ecotoxicology, University of Leuven, Leuven, Belgium; Laboratory of Evolutionary Stress Ecology and Ecotoxicology, University of Leuven, Leuven, Belgium; School of Biological Sciences, University of Aberdeen, Aberdeen, United Kingdom; Department of Zoology, Faculty of Biology, University of Seville, Seville, Spain; Instituto de Ecología A.C., Xalapa, Veracruz, México; Laboratory of Evolutionary Stress Ecology and Ecotoxicology, University of Leuven, Leuven, Belgium

**Keywords:** thermal plasticity, thermal evolution, range expansion, DNA methylation, warming

## Abstract

Due to global change, many species are shifting their distribution and are thereby confronted with novel thermal conditions at the moving range edges. Especially during the initial phases of exposure to a new environment, it has been hypothesized that plasticity and associated epigenetic mechanisms enable species to cope with environmental change. We tested this idea by capitalizing on the well-documented southward range expansion of the damselfly *Ischnura elegans* from France into Spain where the species invaded warmer regions in the 1950s in eastern Spain (old edge region) and in the 2010s in central Spain (new edge region). Using a common garden experiment at rearing temperatures matching the ancestral and invaded thermal regimes, we tested for evolutionary changes in (thermal plasticity in) larval life history and heat tolerance in these expansion zones. Through the use of de- and hypermethylating agents, we tested whether epigenetic mechanisms play a role in enabling heat tolerance during expansion. We used the phenotype of the native sister species in Spain, *I. graellsii*, as proxy for the locally adapted phenotype. New edge populations converged toward the phenotype of the native species through plastic thermal responses in life history and heat tolerance while old edge populations (partly) constitutively evolved a faster life history and higher heat tolerance than the core populations, thereby matching the native species. Only the heat tolerance of new edge populations increased significantly when exposed to the hypermethylating agent. This suggests that the DNA methylation machinery is more amenable to perturbation at the new edge and shows it is able to play a role in achieving a higher heat tolerance. Our results show that both (evolved) plasticity as well as associated epigenetic mechanisms are initially important when facing new thermal regimes but that their importance diminishes with time.

## Introduction

Given that global change is confronting many species with new conditions, a pressing question emerges (Kelly, 2019): what processes underlie successful responses to cope with these novel conditions? Phenotypic plasticity is an immediate response that enables individuals to survive under rapid change (Fox et al., 2019). Yet, it might also be limited and associated with costs (Snell-Rood et al., 2018). Moreover, ancestral plasticity in the old environment might not be adaptive in the new environment (Swaegers et al., 2020; Velotta & Cheviron, 2018; Walter et al., 2020). Evolution may be crucially needed to avoid population extinction under new conditions (Gunderson & Stillman, 2015). Nevertheless, even in the latter case, phenotypic plasticity may still play a key role, as it may both slow down or accelerate evolutionary responses to novel conditions (Ghalambor et al., 2015; Kelly, 2019). This interplay between plasticity and evolution is a major, but still highly debated, research topic (Diamond & Martin, 2016; Fox et al., 2019; Pfennig, 2021a).

An understudied mechanism that can contribute to adaptive plasticity in a new environment are environmentally induced epigenetic modifications (e.g., DNA methylation) (Loughland et al., 2021). Especially during the initial phases of exposure to a new environment, these are regarded as important plasticity-generating mechanism to cope with environmental change (Chevin et al., 2022; Ryu et al., 2018; Vannier et al., 2015). Epigenetic modifications can be an independent evolutionary force as they have the potential to be transmitted across generations independently from the genome (Anastasiadi et al., 2021). However, empirical evidence to back up these theoretical ideas is still scarce (McGuigan et al., 2021). Notably, it has been hypothesized that in later phases of adaptation natural selection is expected to act on the epigenetic variation underlying plasticity, which can potentially canalise selected phenotypes (Pfennig, 2021a).

Widespread ‘natural experiments’ are now taking place whereby many species expand their range and thereby encounter new thermal conditions that deviate from those of their source region (Bujan et al., 2021; Lancaster, 2016). Range expansions allow to study rapid evolution in nature where the source populations in the core region can be considered as a proxy for the ancestral state of the established populations in the new thermal regime (Carbonell & Stoks, 2020; Rohner & Moczek, 2020; Santi et al., 1983). When combined with common-garden experiments, this is a powerful setting to disentangle the plastic and evolutionary mechanisms shaping responses to new thermal regimes (Swaegers & Koch, 2022). Epigenetic mechanisms can play an important, but largely unexplored role during range expansion (Hanson et al., 2020; Hawes et al., 2018; Sarma et al., 2021). Because range expansion is initially often associated with low-genetic diversity, epigenetic variation could be crucial for the success of species to establish and maintain populations in a new thermal regime, especially during the early stages of a range expansion (Marin et al., 2020). While not experimentally tested in the context of range expansion, epigenetic responses and particularly DNA methylation have been linked to population differences in the ability to cope with extreme temperature stress. For example, in the pacific oyster, seagrass and rapeseed hypermethylation during heat stress occurs stronger in heat-sensitive populations than in heat-tolerant populations (Entrambasaguas et al., 2021; Gao et al., 2014; Wang et al., 2021).

Given the clear directionality of the range expansion process it is possible to identify newly founded populations that may shed light on the ‘intermediate’ phases toward the ‘final’ thermal adaptation as seen in the older more evolved populations when the range front progresses. Studies on range expansion can therefore provide unique temporal insights in the still poorly documented process of thermal evolution ‘in action’ in natural populations and the possible role of ancestral plasticity and epigenetic mechanisms in this process. Understanding whether and how plasticity and epigenetic mechanisms are involved in the initial phases of exposure to a new thermal regime during range expansion, what their relative role is in thermal adaptation and how their contribution may shift when more time passes in the new thermal regime is of major importance to understand and predict responses, hence, the fate of populations under global change (McGuigan et al., 2021; Pfennig, 2021b).

Here, we test the role of plasticity and associated epigenetic mechanisms throughout the process of thermal evolution during a range expansion toward a warmer region in an aquatic insect. We make use of the range expansion of the damselfly *Ischnura elegans* from Southern France into Spain that started in the 20th century (Sánchez-Guillén et al., 2013). The exact cause of this southward expansion is unknown. Possibly, the construction of artificial water bodies used for irrigation in Spain (Duarte et al., 2014), which largely coincided with the period of expansion, was one driving factor. Such water bodies may indeed cause the colonization of new areas by the species, as observed in France (Ruggiero et al., 2008). This southward range expansion is associated with the invasion of a region with higher summer water temperatures and more frequent and intense heat waves (Swaegers et al., 2022). Southern French core populations are not well adapted (e.g., suffer higher mortality) to the warmer thermal regime in Spain (Carbonell & Stoks, 2020). In line with this, range-expanding Spanish populations of *I. elegans* show rapid adaptive evolution of heat tolerance and life history (Carbonell & Stoks, 2020). For life history, it was tested and shown that this was in the direction of the locally adapted *I. graellsii* populations, native to Spain and absent from France (Swaegers et al., 2022). While these studies focused largely on old edge populations that have had multiple decades for thermal evolution to occur, we now in addition studied new edge populations that were exposed only about one decade to the new thermal regime. We thereby address the question how plasticity and epigenetic mechanisms contribute to thermal responses in the initial phases of exposure to a new environment. To assess if thermal responses were adaptive, comparisons were made with the phenotype of the locally adapted native sister species in Spain, *I. graellsii* (Swaegers et al., 2022). This species comparison can be justified as in related coenagrionid damselflies, the voltinism of species with a different overall latitudinal distribution was shown to converge for populations occurring at the same latitude (Nilsson-Örtman et al., 2012), and the voltinism is an important driver of the pace-of-life in *Ischnura* damselflies (Debecker & Stoks, 2019). Moreover, both *Ischnura* species share the same microhabitats and flight period (Wellenreuther et al., 2018; J. Swaegers et al., 2021, pers. obs.).

By performing a common garden experiment we tested whether (a) ancestral plasticity contributed to evolved life history and heat tolerance in old edge populations, (b) epigenetic mechanisms play a role in generating plasticity by evaluating the effect of hyper- and demethylating agents on heat tolerance (Sarma et al., 2020), and (c) their role is more pronounced in new compared with the old edge populations (Marin et al., 2020). As key fitness-related life history traits in damselflies we quantified larval development and growth rates, and mass when entering the final larval stage (which is highly correlated to adult mass in the study species, Tüzün et al., 2020). Development and growth rates are associated with voltinism adaptations, whereby faster rates reflect a higher voltinism (Shama et al., 2011). As in Carbonell & Stoks (2020) we estimated the heat tolerance as the critical thermal maximum (CT_max_), the maximum temperature an organism can endure during acute exposure. This is justified as species and populations with a higher CT_max_ have been shown to occupy ranges with higher temperatures (Alruiz et al., 2022; Sunday et al., 2012; Vorhees et al., 2013). Measures of CT_max_ can hence considered to be ecologically relevant and be informative on the thermal region a species can occupy. We focus on DNA methylation as this has been linked to population differences in the ability to cope with extreme temperature stress (McCaw et al., 2020) and has been shown to causally regulate heat tolerance in the study species (as shown by Areshi et al., 2022). In addition, we tested whether DNA methylation levels are the highest in ancestral and new edge populations (i.e., expected to be heat-sensitive) compared with old edge populations and locally adapted *I. graellsii* populations (i.e., expected to be more heat tolerant) (based on Entrambasaguas et al., 2021; Gao et al., 2014; Wang et al., 2021).

## Methods

### Study populations

**Figure 1.**
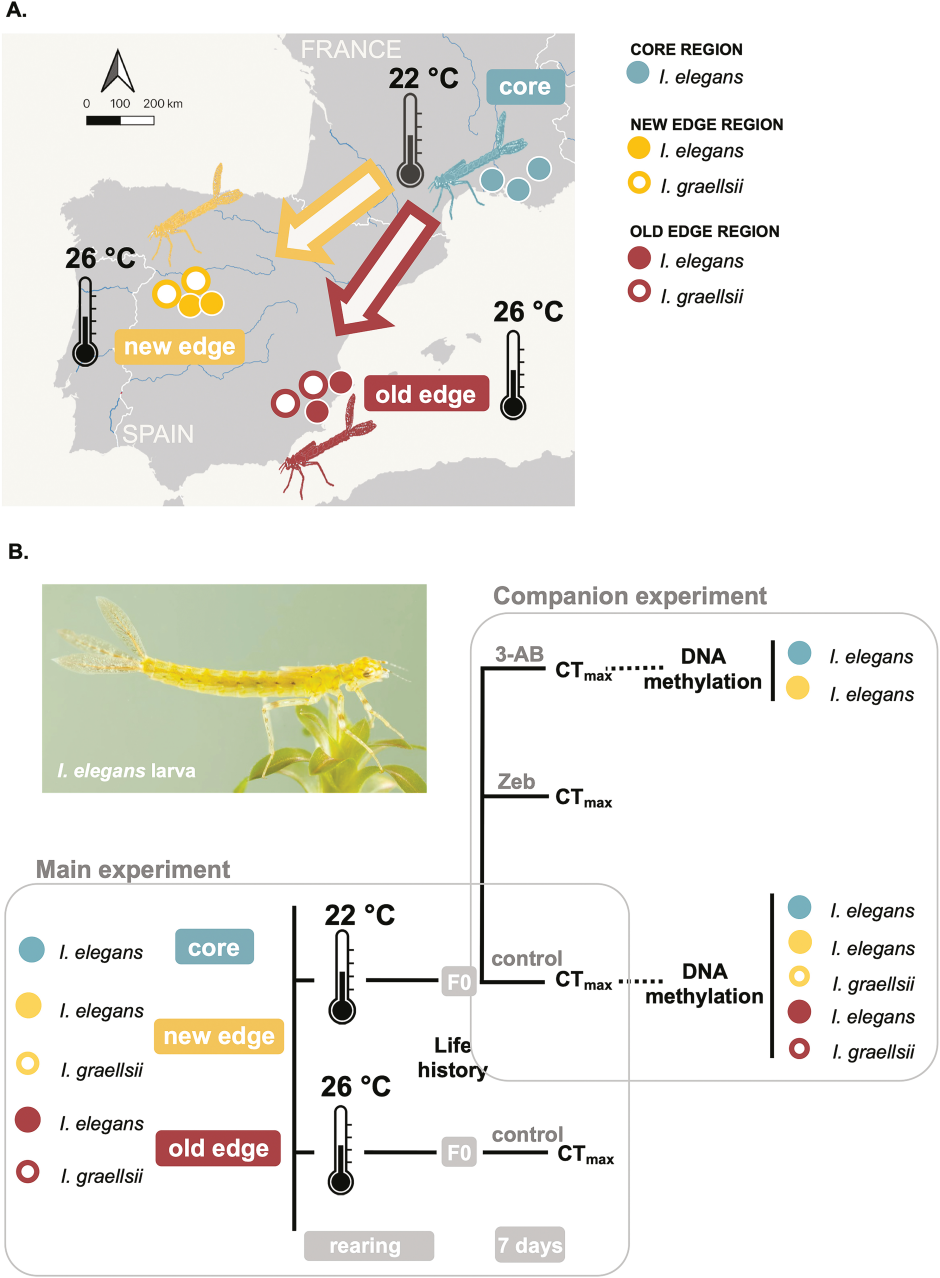
A. Map showing the five population types and 11 populations that were studied to characterize thermal responses during the range expansion of the damselfly *Ischnura elegans*: (i) core populations of *I. elegans* in southern France, the source region of this southward range expansion and a proxy for the ancestral phenotype, (ii) new edge populations of *I. elegans* in the newer range expansion zone in Spain, (iii) old edge populations of *I. elegans* in the older range expansion zone in Spain, and (iv-v) populations of *I. graellsii*, the sister species native to Spain and a proxy for the locally adapted phenotype, in both expansion zones. Depicted are the rearing temperatures which are associated with the thermal regimes encountered for a large portion of the growing season in core and expansion zones (see Supplementary Table S1). B. Scheme of the experimental design. Depicted are the main experiment, which inferred thermal responses in life history and heat tolerance (CT_max_) between population types, and the companion experiment, which inferred drug treatment effects on heat tolerance (CT_max_) and DNA methylation levels between population types. Photo taken by Christophe Brochard.

The focal species, *I. elegans*, expanded its range southward from southern France into Spain in the 20^th^ century (Sánchez-Guillén et al., 2013). We here focus on two range expansion fronts in different regions in Spain where the expansion had a different timing (Figure 1a). In the 1950s, the species colonized the Spanish east coast where it reached its most southern limit in the region of Alicante (Andreu Rubio, 1953). In the 1980s, *I. elegans* colonized Central Spain, (Ocharan, 1987) with its southern limit now reaching the region of Salamanca (M. Rodríguez Esteban, pers. comm.). The first records of *I. elegans* in the Salamanca region date from 2011 (M. Rodríguez Esteban, pers. comm.). Females of *I. elegans* were collected from three core populations (southern France), two edge populations from the more recent expansion zone in Salamanca and two edge populations from the older expansion zone around Alicante (Figure 1A). Females of *I. graellsii* were collected from two populations per region matching the two range expansion zones of *I. elegans*.

Compared with the French populations of *I. elegans*, the Spanish expansion zones of *I. elegans* in both Salamanca and Alicante experience a warmer thermal regime (Supplementary Table S1). In the summer of 2021, females were sampled from all eleven populations and allowed to oviposit in wet filter paper. Wet filter paper with eggs was sent to the laboratory in Leuven where egg hatching occurred. Larvae from 6 to 18 females per population (18–54 females per region; total of 155 females, 1848 larvae) were reared from the egg stage under common-garden conditions.

### Experimental setup

In the main experiment (Figure 1B), larvae from each population type (core, new edge and old edge *I. elegans*, and *I. graellsii* populations matching the two range expansion zones of *I. elegans*) were reared at one of two rearing temperatures: 22 °C and 26 °C. These temperatures represent those of the studied populations during an important part of the larval growth season and allow us to test the evolution of thermal responses associated with range expansion toward a warmer region. Specifically, 22 °C matches the current mean water temperature in southern France from mid-April to mid-September, and hence represents the mean temperature of the core *I. elegans* populations that are the source of the Spanish range expansion. We chose 26 °C as this temperature is often experienced in the edge populations in Spain, but much less so in the core populations in southern France (Supplementary Table S1). The common garden experiment was performed following established protocols (e.g., Van Dievel, Tüzün, & Stoks, 2019). When larvae hatched in the laboratory, they were randomly divided over the two rearing temperatures, and reared individually at this temperature up to the final larval stage (‘F0’).

In a companion experiment (Figure 1B), we imposed three drug treatments to manipulate DNA methylation levels. For budgetary reasons, this was only done at 22 °C. To alter DNA methylation levels, we used two drugs known to affect whole-genome DNA methylation levels: zebularine and 3-aminobenzamide. Zebularine (Zeb) is a nucleoside analog of cytidine which decreases whole-genome DNA methylation levels (Zhou et al., 2002). 3-aminobenzamide (3-AB) is an inhibitor of poly ADP ribose polymerase which increases DNA methylation levels (Zardo et al., 1999). The mode of action of Zeb is similar to 5-azacytidine (5-aza) and 5-aza-2'- deoxycytidine (decitabine), which limit the catalytic activity of DNA methyltransferase by making covalent bonds with it (Zhou et al., 2002). Besides a similar mode of action, Zeb is much more stable resulting in a significantly longer half-life so that it can be used for exposure periods of several days (Baubec et al., 2009). When larvae reared at 22 °C reached the final F0 stage they were exposed for 7 days to either dechlorinated tap water (control treatment), a Zeb solution or a 3-AB solution. The water of each vial was replaced with 50 mL of either solution. A concentration of 65 μM was chosen for both Zeb and 3-AB as a similar concentration of Zeb was used in tadpoles (100 μM, Sarma et al., 2020) and of a related drug in Daphnia (50 μM, Lindeman et al., 2019).

### Life history traits

For all larvae, we estimated three life history traits in the main experiment: development rate, growth rate until F0 and mass at F0. Development rate was quantified as the inverse of development time from egg hatching until moult in the final instar (F0). Growth rate until F0 was calculated as ln (mass start F0)/development time (as in Johansson et al., 2001). In the companion experiment, growth rate during the 7-day drug treatment period was calculated as [ln(final mass) − ln (mass start F0)]/7.

### Thermal tolerance assays

In both experiments, the heat tolerance (critical thermal maximum; CT_max_) was assessed using a dynamic assay optimized for the study species (Lancaster et al., 2015) at day 7 into F0. Sample sizes per population type were as follows: 22°C - control: 80–109; 26°C – control: 26–46; 22°C - Zeb: 31–49; 22°C – 3-AB: 32–60). Note that the set of individuals reared at 22 °C under control conditions are shared between the main and companion experiments. The temperature was ramped up by 0.1 °C per minute until larvae became immobilized. At this temperature, CT_max_, animals lose their motor control due to loss of muscle coordination. Larvae were individually placed in plastic cylinders of 50 mL filled with 12 mL of aged, dechlorinated tap water that were placed in a numbered grid of a thermocycler. Three identical thermocyclers were used that each had the capacity to assay 11 larvae at the same time. The temperature at which each larva became immobilized was assessed. Subsequently, immobilized damselflies were immediately transferred to their matching well to recover for 15 minutes. Individuals that did not recover (i.e., were transferred after their CT_max_), were excluded from the dataset (*N* = 31, 3%). After the assay, larvae were preserved in RNAlater. A subset of larvae (32%) did not get the time to recover after immobilization and were immediately submerged in RNAlater and were used for global DNA methylation measurement (see further). All the larvae were stored at −80 °C.

### Global DNA methylation levels

To assess the effect of 3-AB on methylation levels at CT_max_ in the core and new edge region, we measured global DNA methylation levels in a subset of samples that had undergone the heat tolerance assay in the companion experiment. For budgetary reasons and based on the response patterns in CT_max_, we only tested the effect of 3-AB in core and new edge populations. In addition, we assessed the effect of the population type on DNA methylation levels in the control treatment at CT_max_ for all population types. DNA was extracted using an AllPrep DNA/RNA Mini Kit (Qiagen, 87% of samples) and a DNeasy Kit (Qiagen, 13% of samples). Levels of 5-methylcytosine were quantified using the Global DNA Methylation ELISA Kit of Bio-Connect (Huissen, the Netherlands). For this, 5000 ng of genomic DNA from each sample was used. A 5-methylcytosine standard curve was generated through positive controls ranging from 0.078 µM to 10 µM of 5-methylcytosine to quantify the concentration of methylated DNA in each sample. We analyzed the effect of 3-AB in core and new edge populations (*N* = 54 larvae), and the effect of population type (*N* = 42 larvae, in the absence of 3-AB) on methylation levels in two separate subsets.

### Statistical analyses

Statistical analyses were performed in RStudio version 4.0.2 (R Development Core Team, 2017). For the life history traits, we ran generalized linear mixed-effects models using the package lme4 (Bates et al., 2015), and the package “car” (Fox et al., 2013). F-statistics and corresponding *p* values for fixed effects were estimated using the Kenward–Roger method. We fitted population type (“PopType”), rearing temperature (“RearingT”), drug treatment (if applicable, “DrugTreat”) and all their interactions as fixed effects. In addition, we added population nested in population type, mother nested in population and DNA extraction method (if applicable), as random effects. Sex was added as a cofactor. In the models with heat tolerance as response variable, thermocycler and grid number nested within thermocycler were included as random factors. We ran the models for heat tolerance both with and without mass at the end of the experiment as a covariate. All the models were fitted using restricted maximum likelihood. For each response variable we visually inspected the distribution of residuals to confirm the fit of the univariate model, and accordingly logarithm- or square root-transformed several response variables (see results for details). Significant interactions were further explored using pairwise post hoc comparisons with the “emmeans” function and with correction for multiple testing using the false discovery rate method.

## Results

### Effects of rearing temperature and population type on life history

**Figure 2.**
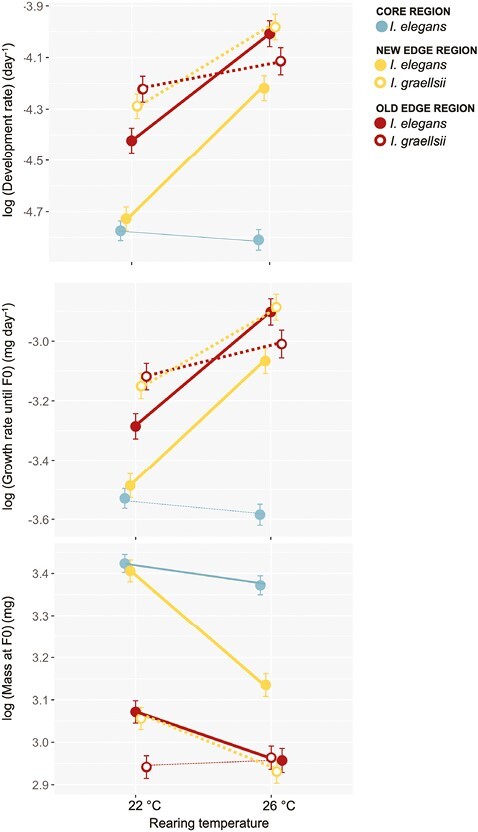
Patterns in life history traits and their thermal plasticity in the five population types associated with the range expansion of *I. elegans*. (A) Development rate until the final larval stage (F0), (B) growth rate until the final larval stage, and (C) mass at the start of the final larval stage. Given are the population type means ± 1 SE and the thermal reaction norms. For population types that responded significantly to temperature the reaction norms were drawn thicker.

Development rate, growth rate until F0, and body mass when entering F0 significantly differed among larvae of the five population types and between larvae reared at the two temperatures. Moreover, for all three life history traits, there was a significant interaction between both terms (Table 1A–C; Figure 2). Core populations did not increase development and growth rates between 22 °C and 26 °C while all other populations showed significantly faster development and growth rates at 26 °C (post hoc tests: all *p* < .05, except French core 22°C vs. 26°C: *p* > .05). The fastening of the pace-of-life at the higher rearing temperature was strongest in the new edge populations (development rate: 11% increase; −1 to 9% in the other population types; growth rate: 12%; −1 to 12% in the other population types). For body mass, the significant interaction between population type and rearing temperature was driven by the new edge populations showing the strongest decrease at 26 °C (−8%; −4 to 1% in the other population types), and only the *I. graellsii* populations from the old edge region showing no significant decrease in body mass at 26 °C (post hoc test: *p* > .05; all other populations: *p* < .05).

**Table 1.** Results of GLMs testing for effects of population type and temperature on (A) development rate, (B) growth rate until F0, and (C) mass at F0 of *I. elegans* in the main experiment.

Life history				
A. log (Development rate)				
	*F*	Df	Df.res	*p*
PopType	31.9	4	6	**<.001**
Temperature	3.4	1	2010	.065
Sex	66.4	1	2013	**<.001**
Temperature × PopType	124.3	4	2001	**<.001**
**B. log (Growth rate until F0)**				
	F	Df	Df.res	*p*
PopType	23.31	4	6	**<.001**
Temperature	9.38	1	1932	**.002**
Sex	28.02	1	1936	**<.001**
Temperature × PopType	113.15	4	1924	**<.001**
**C. log (Mass at F0)**				
	F	Df	Df.res	*p*
PopType	80.58	4	6	**<.001**
Temperature	21.77	1	2040	**<.001**
Sex	373.96	1	2050	**<.001**
Temperature × PopType	75.56	4	2025	**<.001**

*Note*. *p* values <.05 are indicated in bold.

At 22 °C, core and new edge populations showed a slower development rate and weighed significantly less at F0 compared to old edge and *I. graellsii* populations (post hoc tests: all *p* < .05, except French core 22 °C vs. *I. elegans* Alicante 22 °C: *p* = .095) (Figure 2). At 26 °C, core populations showed a slower development and growth rate until F0 compared to the Spanish *I. elegans* and *I. graellsii* populations (post hoc tests: all *p* < .05). New edge populations showed intermediate values for development rate, growth rate, and mass at F0 when reared at 26 °C between the core populations and old edge and the *I. graellsii* populations.

### Effects of rearing temperature and population type on the heat tolerance (CT_max_)

**Figure 3.**
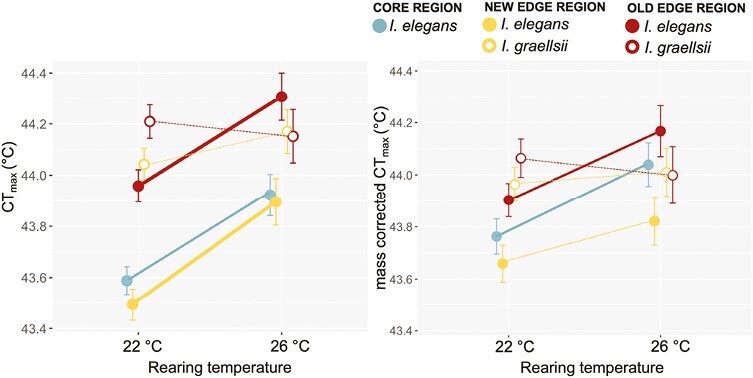
Patterns in heat tolerance and its thermal plasticity in the five population types associated with the range expansion of *I. elegans* in the main experiment: (A) CT_max_ and (B) mass-corrected CT_max_. Given are the population type means ± 1 SE and the thermal reaction norms. For population types that responded significantly to temperature the reaction norms were drawn thicker.

CT_max_ differed among the population types and rearing temperatures, and the effect of rearing temperature differed between population types (Table 2A; Figure 3A). Correcting for mass in the model resulted in similar patterns, except for the interaction term between population type and rearing temperature becoming a trend (i.e., less significant) (Table 2B; Figure 3B). All *I. elegans* populations exhibited greater heat tolerance after rearing at 26 °C, compared to 22 °C (post hoc tests 22 °C vs. 26 °C: all *p* < .05). Instead, *I. graellsii* populations did not differ in heat tolerance between both rearing temperatures (post hoc tests 22 °C vs. 26 °C: all *p* > .05). However, after correcting for mass, significant differences in heat tolerance between rearing temperatures were only observed for core populations and old edge populations (post hoc tests: all *p* < .05).

**Table 2.** Results of GLMs testing for effects of population type and temperature on heat tolerance of *I. elegans* in the main experiment: (A) CT_max_ and (B) mass-corrected CT_max_.

A. CT_max_				
	*F*	Df	Df.res	*p*
PopType	27.92	4	10	**<.001**
Temperature	14.37	1	533	**<.001**
Sex	1.25	1	543	.264
Temperature × PopType	3.54	4	512	**.007**
**B. mass-corrected CT**_**max**_				
	*F*	Df	Df.res	*p*
PopType	3.54	4	12	**.038**
Temperature	9.84	1	536	**.002**
Sex	0.12	1	543	.728
Mass	17.30	1	430	**<.001**
Temperature × PopType	2.23	4	514	.064

*Note*. *p* values <.05 are indicated in bold.

At both rearing temperatures, *I. graellsii* populations and old edge populations displayed the highest heat tolerance (Figure 2A). Differences in heat tolerance among population types decreased at 26 °C (Figure 2A; post hoc tests at 22 °C: all *p* < .05; post hoc tests at 26 °: all *p* > .05).

When larval body mass was included in the model, it showed a significant effect on CT_max_ (Table 2B) with lighter larvae showing a higher heat tolerance (slope = −0.025, SE = 0.006). Correcting for larval body mass resulted in an increase in the least squares mean values of CT_max_ of new edge populations at 22 °C. For new edge populations at 26°C, which were significantly lighter (post hoc test: *p* < .05), correcting for larval body mass in the model resulted in a decrease in the least squares mean value of CT_max_. Differences in heat tolerance between rearing temperatures became non-significant for the new edge populations (post hoc test: *p* > .05). Significant differences in heat tolerance between rearing temperatures were still observed for core populations and old edge populations, after including larval body mass (post hoc tests: all *p* < .05).

### Effects of de/hyper-methylating agents and population type on heat tolerance (CT_max_)

We quantified the effects of Zeb and 3-AB on mortality and growth rate during the 7-day exposure period to assess the potential stressful effects of these drugs. Mortality did not differ among the population types and the drug treatments (Supplementary Table S2). Growth rate, however, differed among the drug treatments (Supplementary Table S3, Supplementary Figure S1). Zebularine decreased growth rate compared to the control (*p =* .021), while for 3-AB this was only a trend (*p =* .058) (Supplementary Tables S2, S3, Supplementary Figure S1).

**Figure 4.**
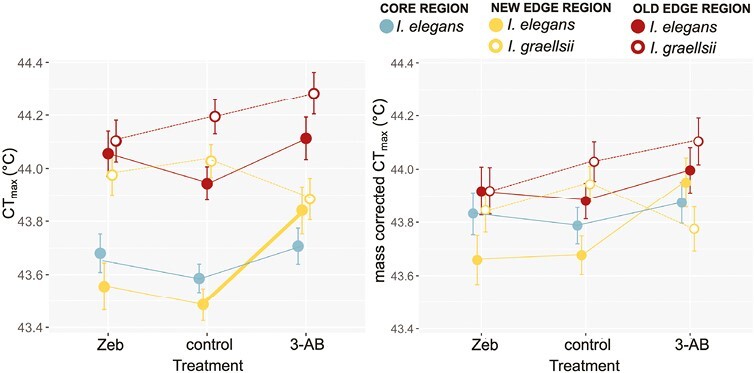
Effect of the drug treatment on the heat tolerance in the five population types associated with the range expansion of *I. elegans* in the companion experiment: (A) CT_max_ and (B) mass-corrected CT_max_. Given are the population type means (large symbols) ± 1 SE and the thermal reaction norms. For population types that responded significantly to temperature the reaction norms were drawn thicker.

In the subset of larvae reared at 22 °C where the drug treatment was imposed, CT_max_ differed among the population types and the drug treatment had a different effect among the population types (Table 3A, Figure 4A). This interaction was mainly driven by a higher heat tolerance of the new edge populations in the AB-3 treatment compared to the heat tolerance of these populations in the control and Zeb treatments (post hoc tests: *p* < .05). Zeb, on the other hand, had no effect on heat tolerance (all post hoc tests: *p* > .05). The heat tolerance of the new edge populations exposed to AB-3 was statistically indistinguishable from native *I. graellsii* in that region (post hoc test: *p* > .05), while it was lower in control conditions (post hoc test: *p* < .05). When new edge populations were exposed to AB-3 also the difference in CT_max_ with *I. elegans* and *I. graellsii* of the old edge region became smaller. When larval body mass was included in the model, it showed a significant effect on CT_max_ (Table 3B) with lighter larvae showing a higher heat tolerance (slope = −0.030, SE = 0.005). When corrected for body mass, the Drug treatment × Population type interaction was only a trend (Table 3B, Figure 3B). No significant differences were found in the post hoc comparisons between the treatments. The body mass correction resulted in an increase in heat tolerance for the heavier (larger) core populations and new edge populations, and a decrease in heat tolerance for the lighter old edge populations and *I. graellsii* populations, thereby diminishing overall CT_max_ variation.

**Table 3.** Results of GLMs testing for effects of population type and drug treatment on heat tolerance of *I. elegans* in the companion experiment: (A) CT_max_ and (B) mass-corrected CT_max_.

A. CT_max_				
	*F*	Df	Df.res	*p*
PopType	10.05	4	48	**<.001**
DrugTreat	1.47	2	788	.230
Sex	0.10	1	788	.754
DrugTreat × PopType	2.32	8	776	**.018**
**B. mass-corrected CT**_**max**_				
	F	Df	Df.res	*p*
PopType	2.57	4	52	**.049**
DrugTreat	0.67	2	788	.513
Sex	1.98	1	787	.160
Mass	28.96	1	631	**<.001**
DrugTreat × PopType	1.89	8	773	.059

*Note*. *p* values <.05 are indicated in bold.

### Global DNA methylation levels

**Figure 5.**
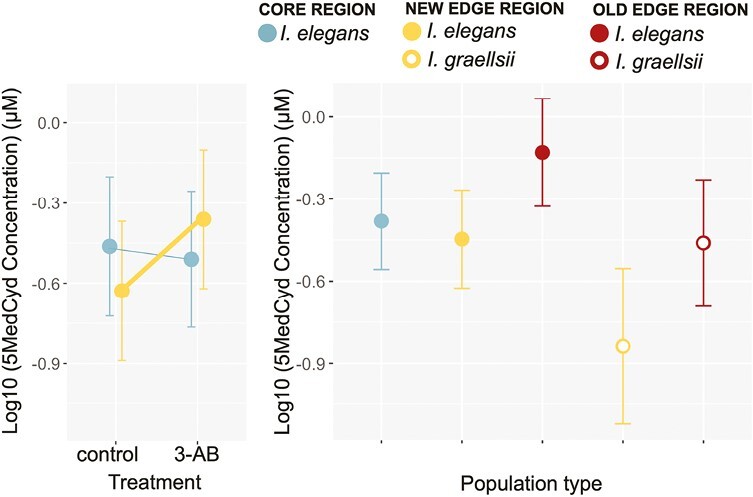
Patterns in DNA methylation levels in (A) core and new edge population types as a function of the imposed drug treatment and (B) all population types in the control treatment. Given are the population type means (large symbols) ± 1 SE and the reaction norms. For population types that responded significantly to the drug treatment the reaction norms were drawn thicker. Note that as the model associated with panel B has more data to estimate the random factors in the model, the means of the core and new edge control individuals have “gravitated” toward the grand mean effects. The estimated means of the control treatment do therefore not match those of the left panel.

Global DNA methylation levels in the control samples did not differ among the population types (*F*_1,13_ = 1.54, *p* = .292; Figure 5). Core populations and new edge populations responded differently to the 3-AB treatment as indicated by the significant Population type ×Drug treatment interaction on global DNA methylation levels (*F*_1,18_ = 6.68, *p* = .019; Figure 5). Contrary to the core populations, the new edge populations showed the expected increase in global methylation levels in the 3-AB treatment (*p*<.05). These patterns remained when correcting for body mass (*F*_1,20_ = 4.41, *p* = .049).

## Discussion

Both phenotypic plasticity and rapid evolution can contribute to the colonization and adaptation to novel environments. Our results suggest that (i) during the initial phases of exposure to a warmer thermal regime during range expansion, adaptive responses were mainly achieved through (evolved) plasticity in life history and heat tolerance while during the later stages also constitutive evolution added to this response. (ii) DNA methylation machinery is less canalized and more amenable to perturbation during the initial phase of exposure to a warmer thermal regime during range expansion and could play a role in achieving a higher heat tolerance. As offspring of field-collected mothers were used in this study, it is not possible to completely rule out a contribution of maternal effects when interpreting phenotypic shifts between population types as the result of evolution. Each model, however, included the identity of the mother as a random factor to control for both maternal genetic and environmental effect variation within populations (Swaegers et al., 2022).

### Plastic and evolved responses in life history and heat tolerance

While both new and old *I. elegans* edge populations showed convergence towards the native sister *species I. graellsii* in both life history traits and thermal tolerance at the warmer rearing temperature, there were clear differences in how they realized this convergence. On the one hand, the new edge populations from the region of Salamanca showed evolution of plasticity for life history traits in response to a higher rearing temperature, thereby showing a partial convergence of life history towards the phenotype of the native *I. graellsii* populations. In addition, results suggested that besides ancestral plasticity (i.e., plasticity in the core region) a higher heat tolerance in response to a higher rearing temperature was at least partially caused by evolution of plasticity of body size. On the other hand, old edge populations showed full convergence towards the locally adapted *I. graellsii* populations through both evolved plasticity and constitutive evolution of life history traits in response to a higher rearing temperature. French core populations had a lower development rate as well as a lower growth rate at both rearing temperatures, thereby showing flat thermal reaction norms for these life history traits. Carbonell & Stoks (2020) and Swaegers et al., (2022) observed the same results for the growth rate of *I. elegans* larvae from the French core and Spanish edge regions at temperatures ranging from 16 °C to 28 °C. This supports the view that evolution of plastic responses may facilitate range expansions of species into a new environment (Lande, 2009 & 2015; Rohner & Moczek, 2020; Thibert-Plante & Hendry, 2011). Moreover, these observed (evolved) plastic responses in life history traits suggest that Spanish *I. elegans* populations can obtain a greater voltinism, hence complete more generations per year. This is in line with the general pattern of a faster development in insects of lower latitudes in Europe which generally are exposed to higher temperatures (Zeuss et al., 2017). Since a multivoltine strategy is more likely to succeed in warmer climates and a higher voltinism shortens the time frame available for development, it seems that a faster pace-of-life of new edge populations in response to a higher rearing temperature is adaptive (Hill et al., 2021; Nygren et al., 2008; Shama et al., 2011). This pattern further suggests that at warmer temperatures these populations would gain high compound interest benefits, hence benefit from the ability to complete multiple generations when reproduction is restricted to a particular time window (Angilletta et al., 2004; Fischer & Fiedler, 2002). In addition, edge populations showed convergence in their pace-of-life toward the locally adapted *I. graellsii* populations at 26 °C. The smaller body mass when reaching the final larval F0 stage at the higher rearing temperature that was achieved through plasticity (new edge populations) or evolution (old edge populations) is in line with the temperature-size rule which states that animals get smaller at higher temperatures. This rule is often explained by the observation that development rate increases more than growth rate when temperature increases (Rubalcaba et al., 2020; Zeuss et al., 2017), although here increases in these traits were similar in both edge populations.

For heat tolerance (measured as CT_max_), the new edge populations showed a similar thermal response as the core populations and did not converge toward the populations of the native *I. graellsii*. This suggests that heat tolerance in the new edge populations did not evolve but remained ancestral. Instead, old edge populations showed partial convergence in heat tolerance toward the locally adapted *I. graellsii* populations through both ancestral plasticity and constitutive evolution. A plastic increase in heat tolerance at 26 °C was observed in both the core and new edge regions. For the core region this was still the case when heat tolerance was corrected for body mass, but not in the new edge region. This suggests that new edge populations reared at 26 °C at least partly acquired an increased heat tolerance through a decrease in body mass. In a meta-analysis by Leiva et al. (2019), it was found that heat tolerance generally increases with decreasing body size in water-breathing animals. We note that the CT_max_ assay was started from the individual’s rearing temperature of 22 °C or 26 °C. This can lead to “artificial” higher CT_max_ values in animals reared at 26 °C (as these are for a shorter time exposed to the increasing temperatures in the CT_max_ trials), but is not expected to bias differences in plasticity in CT_max_ driven by rearing temperature between the different population types. Moreover, this potential of artificial “inflation” of CT_max_ seems limited in our study as *I. graellsii* individuals did not reach a higher CT_max_ when reared at 26 °C compared with 22 °C.

Taken together, our study suggests an important role for both ancestral (heat tolerance) and evolved (life history) plasticity during initial exposure to a new thermal regime. Evolved plasticity in life history and thermal (cold) tolerance has also been detected and suggested to be adaptive at the recent northward range expansion front of a butterfly (Neu & Fischer, 2022). Fine-tuned evolved plastic responses might be a general response to at least initially cope with new environments during range expansion (Lande, 2015). Our results, however, also show that constitutive evolution gains importance when exposure to a new thermal regime persists. Besides plasticity, also constitutive evolution contributed to the partial convergence in life history and heat tolerance of old edge population toward the locally adapted *I. graellsii* population. Potentially, life history and thermal tolerance evolved through the process of genetic accommodation (Levis & Pfennig, 2019). Rapid changes, through both evolved plasticity and constitutive evolution, in these traits could have enabled *I. elegans* to expand their range in Spain as in general can be predicted (Diamond, 2018). Future experiments in this study system would benefit from adding more natural temperature treatments (e.g., daily temperature fluctuations) instead of constant rearing temperatures as patterns of thermal adaptation may become more apparent under such conditions (Kingsolver & Gomulkiewicz, 2003; for the study species: Verheyen & Stoks, 2019), and the interplay between plasticity and evolution might depend on how the environment changes (Vinton et al., 2022).

### Contribution of DNA methylation to heat tolerance

Our results suggest that epigenetic changes are more inducible, and the contributions to thermal tolerance more pronounced in acquiring heat tolerance during the early stage of the range expansion. In invertebrates both promoter and gene bodies can be (de)methylated upon exposure to new conditions, and this can both silence or promote gene expression (Asselman et al., 2017). One way to explain the increase of heat tolerance in the larvae of the new edge region when exposed to 3-AB is that hypermethylation silenced genes associated with physiological “business-as-usual” processes, and therefore more energy could be allocated to cope with heat. As 3-AB did not significantly reduce mortality nor growth rate, any stressful effects of 3-AB itself are likely limited. Upregulation of energetically costly stress proteins has been demonstrated in response to heat stress in this study species, and trade-offs between stress response and “business as usual” functions tend to be more pronounced in (northward) expanding than in core populations (Lancaster et al., 2016). In line with this idea, previous work found that decreased thermal tolerance in methylation-inhibited *I. elegans* (i.e., after treatment with Zebularine) near the northern range expansion front in Great Britain was associated with failure to downregulate genes associated with growth, development, and metabolism (Areshi et al., 2022). Interestingly, 3-AB exposure delayed reproduction in Siberian hamsters (Stevenson & Prendergast, 2013) and decreased fecundity in beetles (McCaw et al., 2022, in review), suggesting that shifts in DNA methylation can indeed be a general mechanism to shift energy allocation. In sticklebacks, hypermethylation was more common than hypomethylation in response to warming, indicating that hypermethylation might be regulating thermal responses through gene (in)activation (Metzger & Schulte, 2017). It should be noted though that invertebrate responses to hypermethylation are not expected to be the same as in vertebrates (Glastad et al., 2011). A study of Dai et al. (2017) found that temperature-stressed whiteflies had an increased mortality when they were fed with BtDnmt1 dsRNA which decreases DNA methylation levels through down-regulating DNA-methyltransferase 1. Although, in our study, and in contrast to results from the northern range margin in *I. elegans* (Areshi et al., 2022), the demethylating agent Zeb had no effect on heat tolerance, it shows that DNA methylation processes can be involved in coping with thermal stress in invertebrates. Although we found upward shifts in the means of 3-AB treated individuals of the new edge region in both CT_max_ and methylation levels, we could not detect a significant correlation between methylation levels and CT_max_ at the individual level; neither overall or within the new edge populations (Supplementary Materials). This may be due to the low variation in CT_max_ values within groups (Figure 3) and/or suggest that a change in DNA methylation is more important for shaping this trait. However, without information on baseline methylation levels at the level of the individual prior to the drug treatment, this hypothesis cannot be tested with the current dataset. An alternative explanation that instead of the methylation level, the exposure to the drug itself shifted CT_max_ seems unlikely. We would then expect a similar effect of the drug treatment on CT_max_ in all regions. Moreover, xenobiotics, if anything have been shown to have adverse effects on CT_max_ (Moe et al., 2013), while we here instead found that 3-AB increased CT_max_ in the new edge populations.

One way to explain the differential effect of 3-AB on global methylation levels and heat tolerance between the new edge region and French core *I. elegans* could be that range expansion into a new thermal regime led to a higher epigenetic potential (i.e., capacity for phenotypic plasticity via epigenetic modifications (Kilvitis et al., 2017)). In a study by Hanson et al., (2022), a higher number of CpG sites was found in introduced populations compared to native populations of house sparrows. In general, gaining CpG sites corresponds to an increased potential for methylation to occur and hence can be considered as a form of epigenetic potential (Feinberg & Irizarry, 2010). If indeed the number of CpG sites increased during range expansion of *I. elegans*, the effect of 3-AB on heat tolerance might have been stronger as 3-AB allowed DNMT1 to methylate previously unavailable sites (Zardo et al., 1999). A higher epigenetic potential in the new edge region of *I. elegans* might have been selected for as it allows for flexible modulation of gene expression and therefore the generation of adaptive responses to a new environment (Hanson et al., 2022; Levis & Pfennig, 2019). Irrespective of the underlying mechanism, the ability of larvae in the new edge region to plastically respond to warming in life history compared to those in the French region indeed suggests a history of selection for flexible regulation.

There was no significant increase of heat tolerance in the 3-AB treatment in the old edge region. As populations from this region had more time for genetic refinements and canalization of plastic responses to a warmer thermal regime, it might be that selection for flexible regulation through epigenetic mechanisms diminished, and that a higher heat tolerance has been genetically anchored through time as suggested by theory (Pfennig, 2021a). As shown in our results, responses to a new thermal regime were partly driven by constitutive evolution toward the phenotype of *I. graellsii* in this region.

## Conclusions

Our natural study system allowed to investigate how responses to a new thermal regime evolve and whether trait values converged to those of a locally adapted sister species. Our results show that both ancestral and evolved plasticity as well as epigenetic mechanisms are initially shaping adaptive responses to the new thermal regimes. The presence of constitutive evolution, on the top of plastic responses, in the old edge populations suggests canalization of these plastic responses through the process of genetic accommodation. In addition, our results suggest that hypermethylation contributes to heat stress responses, potentially as a mechanism to re-allocate energy toward heat-coping mechanisms. This is, to our knowledge, one of the first studies experimentally showing that epigenetic mechanisms can be implicated in coping with heat in natural populations, and that these epigenetic responses are presumably replaced by genetically anchored adaptations in time.

## Supplementary Material

qrac007_suppl_Supplementary_Material

## Data Availability

Data have been deposited in the Dryad Digital Repository (https://doi.org/10.5061/dryad.qfttdz0mw).
