# Supplementary material for: Plasticity and associated epigenetic mechanisms play a role in thermal evolution during range expansion
Source: Evol Lett. 2023 Jan 31;8(1):76–88. doi: 10.1093/evlett/qrac007 (PMC10872138; doi:10.1093/evlett/qrac007)
Supplement: qrac007_suppl_Supplementary_Material [file qrac007_suppl_supplementary_material.pdf]

Supplemental Information for:

Plasticity and associated epigenetic mechanisms play a role in thermal evolution during range expansion

Janne Swaegers, Simon De Cupere, Noah Gaens, Lesley T. Lancaster, José A. Carbonell, Rosa A. Sánchez Guillén, Robby Stoks

METHODS

Water temperature estimation

The Lake Model Flake (2016), a freshwater lake model capable of parametrizing lake temperatures (www.flake.igb-berlin.de), was used to estimate long-term water temperatures from mid-April to mid-September (spanning an important part of the larval growth period) in all population types. An average water depth of 1 m and turbidity of <0.5 m transparency were chosen to parameterize the model, which reflect the typical habitat of *I. elegans* and *graellsii* larvae: shallow, eutrophic and mesotrophic ponds (Dijkstra, 2006). For water bodies inhabited by *I. elegans* it has been validated that average water temperatures estimated with this model closely match the actual water temperatures (Table S1 in Dinh Van et al., 2014).

RESULTS

Mortality and growth rate during F0

Mortality did not differ among the population types and the drug treatments (Table S1). Growth rate, however, differed among the drug treatments (Table S2, Figure S1). AB-3 and Zebularine both decreased growth rate during the 7-day exposure to the drug treatment compared to the control, yet this was only a trend for AB-3 (P = 0.058 for AB-3; P = 0.021 for Zebularine).

Table S1. Characterization of the population types with their study populations.

**Population Type**	**Location**	**Lat.**	**Long.**	**#Days >26 °C**	**mean water temperature (mid-April – mid-September)**
I. elegans France	St. Martin de Crau	43.6	5.0	24	22.1
I. elegans France	Bassin de Réaltor	43.5	5.3	31	22.4
I. elegans France	La Durance	43.7	5.7	31	22.4
I. elegans Spain new	Alba de Tormes	40.8	-5.5	66	23.7
I. elegans Spain new	Torrejón de Alba	40.8	-5.5	66	23.7
I. graellsii Spain new	Ermita de San Bricio	41.0	-5.3	66	23.7
I. graellsii Spain new	Las Torres	40.9	-5.7	66	23.7
I. elegans Spain old	El Hondo	38.2	-0.8	79	24.8
I. elegans Spain old	Oliva-Pego	38.9	-0.1	79	24.8
I. graellsii Spain old	Balsa Ganadera	37.9	-1.9	79	24.8
I. graellsii Spain old	Albacete	38.8	1.4	71	24.7

Table S2.

**Mortality during F0**			
	Chisq	Df	*P*
PopType	4.76	6	0.575
DrugTreat	0.77	3	0.856
Sex	0.15	2	0.929
DrugTreat × PopType	4.88	8	0.770

Table S3.

**Growth rate during F0**				
	F	Df	Df.res	*P*
PopType	2.85	4	10	0.080
DrugTreat	6.71	2	1477	**0.001**
Sex	1.91	1	1494	0.167
DrugTreat × PopType	1.23	8	1468	0.276

Figure S1.

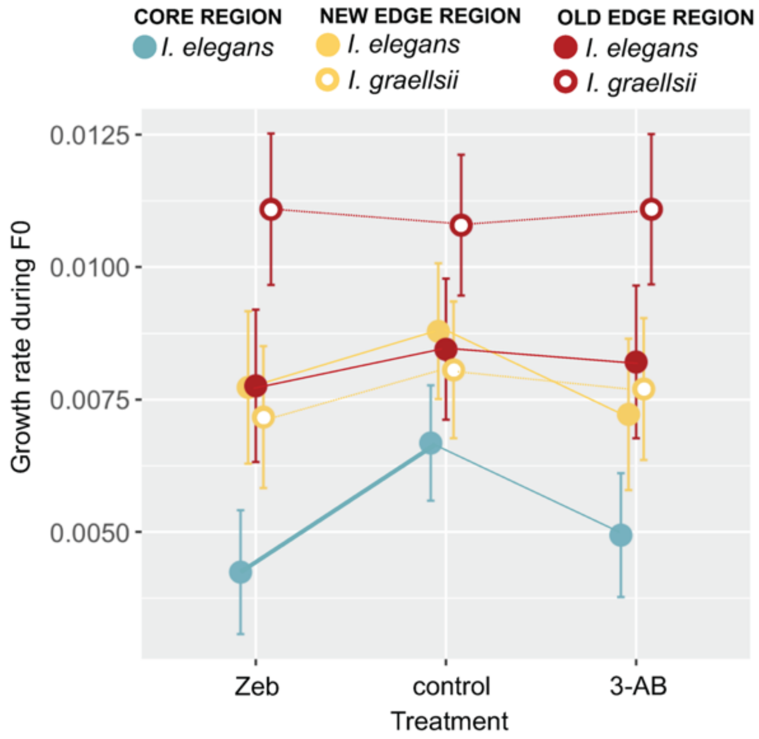

Effect of methylation levels on CT_{max}

There was no significant effect of methylation levels on CT_{max} in the full dataset ($F_{1,54}$=0.85, $P > 0.05$). Also, for the subset of the larvae of the new edge region (as the epigenetic mechanisms were found to be operating in individuals from that region), no effect of the methylation levels on CT_{max} was found although the relationship was slightly positive (slope: 0.08±0.28; $P > 0.05$).
